# Electric Horticulture

**Published:** 1882-06

**Authors:** 


### ELECTRIC HORTICULTURE.

The Abbé Moigno says that the results obtained at the French Palace of Industry in the experiments with electric light in conservatories were not favorable. The naked rays were found, as discovered previously by Dr. Siemens, to be injurious to plants, and when passed through glass globes, did not appear to effect them. The Abbe finds no proof that nocturnal illumination is beneficial to plants.

